# Comment on ‘HER2 overexpression predicts pathological T2 stage and improved survival in de novo muscle-invasive bladder cancer after immediate radical cystectomy: a retrospective cohort study’

**DOI:** 10.1097/JS9.0000000000001527

**Published:** 2024-05-03

**Authors:** Yingping Liu, Xingxing Zhang, Panfeng Shang

**Affiliations:** a The Second Clinical Medical College of Lanzhou University, Lanzhou University; b Department of Urology, Institute of Urology, Key Laboratory of Urological Diseases in Gansu Province, Gansu Nephro-Urological Clinical Center, Lanzhou University Second Hospital, Lanzhou, Gansu, People’s Republic of China

*Dear Editor*,

I read with great interest the recent study by Kim *et al*.^1^ published in the *International Journal of Surgery*, which explores human epidermal growth factor receptor type 2 (HER2) status in de novo muscle-invasive bladder cancer (MIBC). The study offers valuable insights that HER2 overexpression predicts pathological T2 stage and improved survival in de novo muscle-invasive bladder cancer after immediate radical cystectomy (RC). The results may help to develop guidelines for the treatment of MIBC and thus improve the prognosis of patients. Despite the strengths of this study, several underlying concerns require further elucidation.

Firstly, the patient’s surgery-related information needs to be further detailed, including the patient’s duration of surgery and postoperative complications^2,3^. To a certain extent, the absence of this part will compromise the integrity of the research results. Therefore, it is recommended that more surgery-related information about patients be provided and that these factors be accounted for in data analysis.

Secondly, the conclusion that HER2 improves survival needs further justification. Both in all patients and in the RC subgroup, univariate analysis, multivariate analysis, and Kaplan–Meier survival curves showed that age less than 70 years and HER2 status were favorable prognostic factors. In this scenario, a reasonable assumption would be that the observed differences in the median survival period between the two groups may not be solely attributable to the HER2 status but rather influenced by the age, which greatly compromises the reliability and accuracy of the conclusions drawn in this study.

Thirdly, it is essential to compare the performance of HER2 with existing targets that are accepted in this field. DNA damage repair genes such as ATM, RB1, and FANCC are identified biomarkers^1^. Therefore, studies could incorporate measurements of the expression of these biomarkers to compare with the predictive effect of HER2. By comparing the performance of HER2 with these biomarkers, readers will be able to better understand its strengths and limitations, as well as its potential applications in clinical practice.

Fourthly, the interpretation of HER2-immunohistochemistry (IHC) staining results were based on clinical practice guidelines for HER2 detection in breast cancer. Because of the lack of clinical consensus on HER2-IHC staining results in bladder cancer, the clinical practice guideline used was from breast cancer, which would have impaired the reliability of the study’s findings. Therefore, we propose to discuss in detail the similarities and differences in disease characteristics and IHC staining profiles between bladder and breast cancers to demonstrate the feasibility of drawing on clinical practice guidelines for breast cancer to interpret HER2-IHC staining results in bladder cancer. In addition, the operator of the HER2-IHC staining operation and interpretation of the results were not described in detail. Differences in analytical performance and accuracy of the HER2 assay are observed among operators (e.g. medical oncologists, pathologists, surgeons, and radiation oncologists). It is necessary to provide the operator of the operation and interpretation to assess potential bias and to facilitate the replication of the study by other researchers to validate the conclusions.

In conclusion, this study contributes to the literature on guidelines for treating MIBC. In addition to the limitations mentioned above, the relatively small sample size of this study may limit the generalizability of the findings. I believe the quality of this article would improve if the concerns mentioned above were addressed.

## Ethical approval

Not applicable.

## Consent

Not applicable.

## Sources of funding

This work was supported by the Cuiying Scientific Training Program for Undergraduates of The Second Hospital & Clinical Medical School, Lanzhou University (CYXZ2022-18).

## Author contribution

All authors read and approved the final version of the manuscript.

## Conflicts of interest disclosure

The authors declare no conflicts of interest.

## Research registration unique identifying number (UIN)

1. Name of the registry: not applicable.
2. Unique identifying number or registration ID: not applicable.
3. Hyperlink to your specific registration (must be publicly accessible and will be checked): not applicable

## Guarantor

Yingping Liu.

## Data availability statement

No data was generated during the writing of this study.

## Provenance and peer review

Not applicable.
